# Robotic navigation with deep reinforcement learning in transthoracic echocardiography

**DOI:** 10.1007/s11548-024-03275-z

**Published:** 2024-09-20

**Authors:** Yuuki Shida, Souto Kumagai, Hiroyasu Iwata

**Affiliations:** 1 Graduate School of Creative Science and Engineering, Waseda University, Tokyo, 169-8050 Japan; 2 Faculty of Science and Engineering, Waseda University, Tokyo, 169-8050 Japan

**Keywords:** Medical robots, Echocardiography, Robotic ultrasound, Human–robot interaction

## Abstract

**Purpose:**

The search for heart components in robotic transthoracic echocardiography is a time-consuming process. This paper proposes an optimized robotic navigation system for heart components using deep reinforcement learning to achieve an efficient and effective search technique for heart components.

**Method:**

The proposed method introduces (i) an optimized search behavior generation algorithm that avoids multiple local solutions and searches for the optimal solution and (ii) an optimized path generation algorithm that minimizes the search path, thereby realizing short search times.

**Results:**

The mitral valve search with the proposed method reaches the optimal solution with a probability of 74.4%, the mitral valve confidence loss rate when the local solution stops is 16.3% on average, and the inspection time with the generated path is 48.6 s on average, which is 56.6% of the time cost of the conventional method.

**Conclusion:**

The results indicate that the proposed method improves the search efficiency, and the optimal location can be searched in many cases with the proposed method, and the loss rate of the confidence in the mitral valve was low even when a local solution rather than the optimal solution was reached. It is suggested that the proposed method enables accurate and quick robotic navigation to find heart components.

## Introduction

As of 2019, heart disease was the leading cause of death worldwide [1]. However, ultrasonography, including transthoracic echocardiography, is challenging, and physicians are in short supply. To solve these problems, previous studies have proposed a wide range of assistive technologies for ultrasonographic examinations using robots [2–4]. The authors are also working toward automated diagnosis using an echocardiography robot, with the goal of automatically acquiring the basic views necessary for diagnosis [5]. However, the basic view search by an echocardiography robot requires more time than that by a physician. The physician estimates the position of the heart from ultrasound (US) images obtained by applying a probe to a portion of the patient’s chest, and then the probe is pressed against the appropriate position. In contrast, the robot derives the acquisition position of the basic views by acquiring and analyzing a group of US images of the patient’s entire chest during the search process thus, estimating the position of the basic views takes a long time. This problem has been investigated in spinal echocardiography, transesophageal echocardiography, and point-of-care ultrasound (POCUS: simple echocardiography) using deep reinforcement learning (DQN) to realize an efficient search method that is similar to that of a physician [6–8]. Nonetheless, to the best of our knowledge, no research has been conducted specifically on transthoracic echocardiography.

**Fig. 1:**
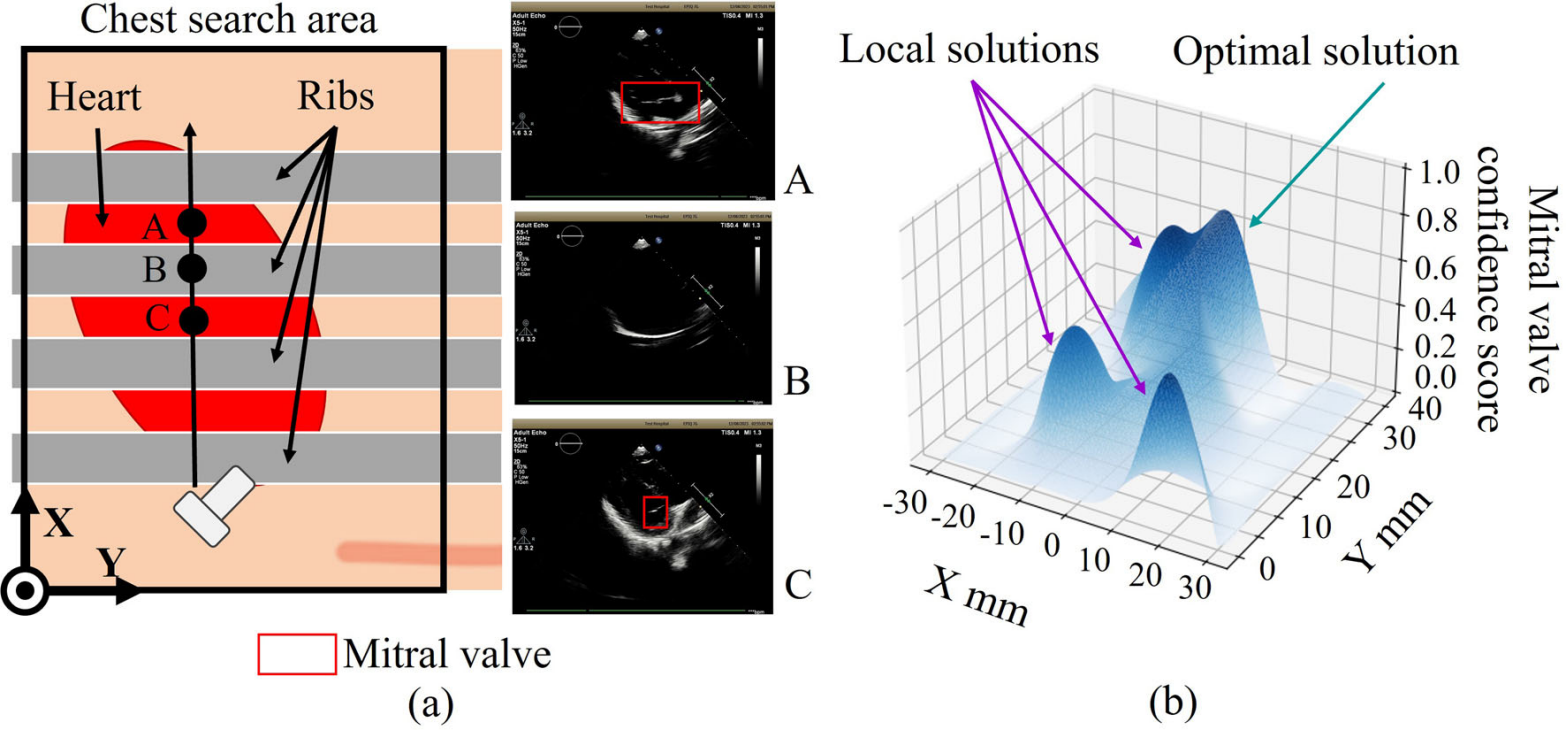
Heart visibility in transthoracic echocardiography: **a** location of heart and ribs, and US images; **b** heart component confidence distribution

When acquiring US images of the heart in transthoracic echocardiography, the lungs and ribs between the chest wall and the heart interfere with the image acquisition process and blur the US images. In particular, the ribs are present at regular intervals along the vertical axis of the body, as shown in Fig. 1a; thus, when the probe is advanced along the X-axis, the heart components are not always clearly visible. Therefore, as shown in Fig. 1b, the clarity of the heart components is intermittent, which results in a local solution when estimating the position of the heart components. In addition, there are individual differences in the position, size, and shape of the heart, and estimating the optimal solution for the location of heart components accurately requires us to discriminate between the local and optimal solutions for individual patients, which is more challenging than in other echocardiographic examinations. The contours of the heart components are not depicted as clearly in the US images at the local solution location as compared to the optimal solution location. Thus, when US images acquired from the local solution location are used to diagnose heart disease, the motion of the heart components cannot be accurately measured, which may lead to misdiagnosis. To address these issues, this paper proposes a system to optimize robot navigation to heart components in transthoracic echocardiography using DQN. The hypothesis of this paper is that by introducing a robotic navigation system using DQN with an ingenious reward in robotic transthoracic echocardiography, it is possible to avoid local solutions and efficiently navigate the probe to the optimal solution where the components of the heart are clearly visible. The purpose of the proposed system is to reduce the number of search paths while maintaining accurate estimation of the heart components.

## Method

### Overview of optimized robotic navigation system for heart components

**Fig. 2:**
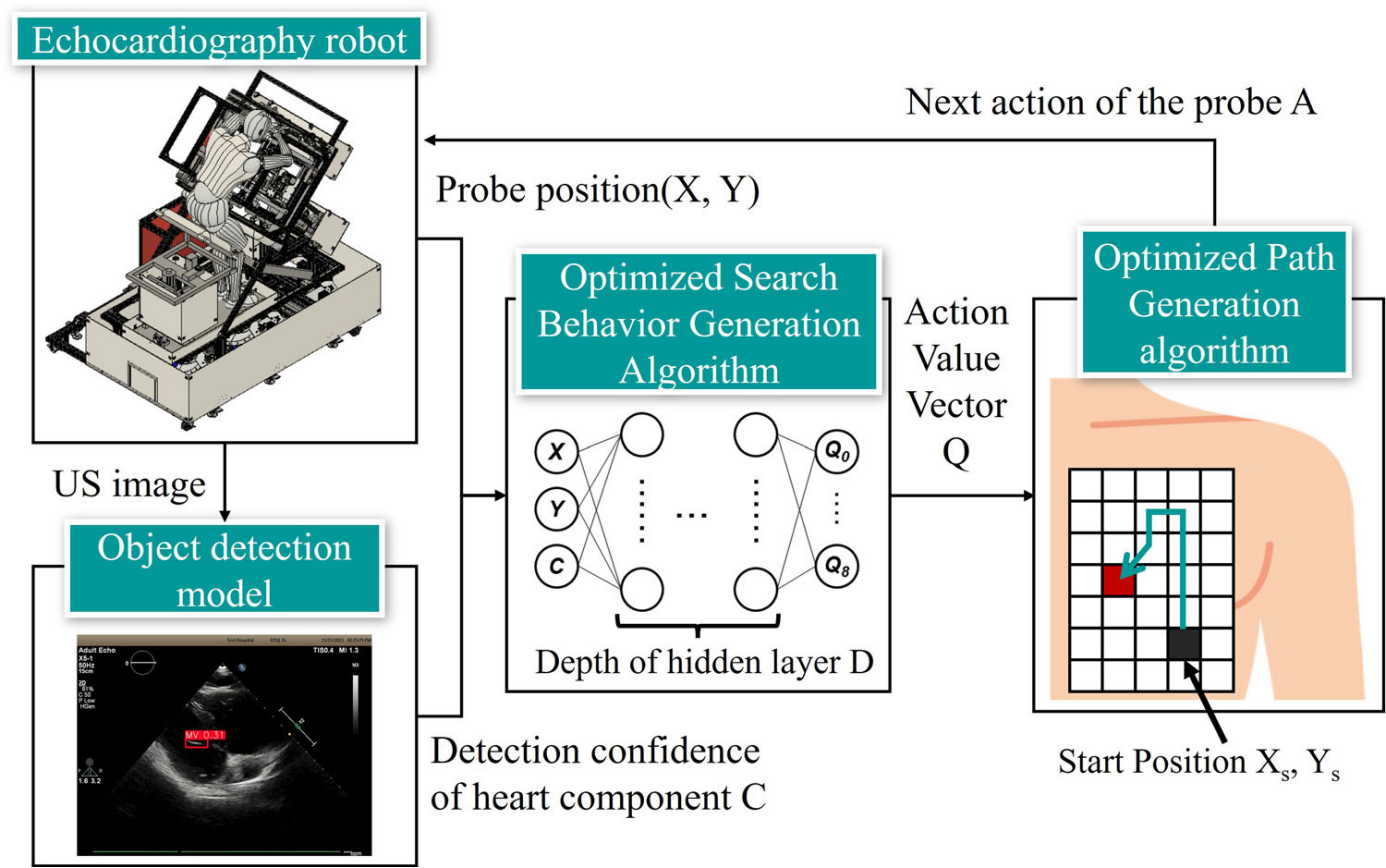
Overview of optimized robotic navigation system for heart components

**Fig. 3:**
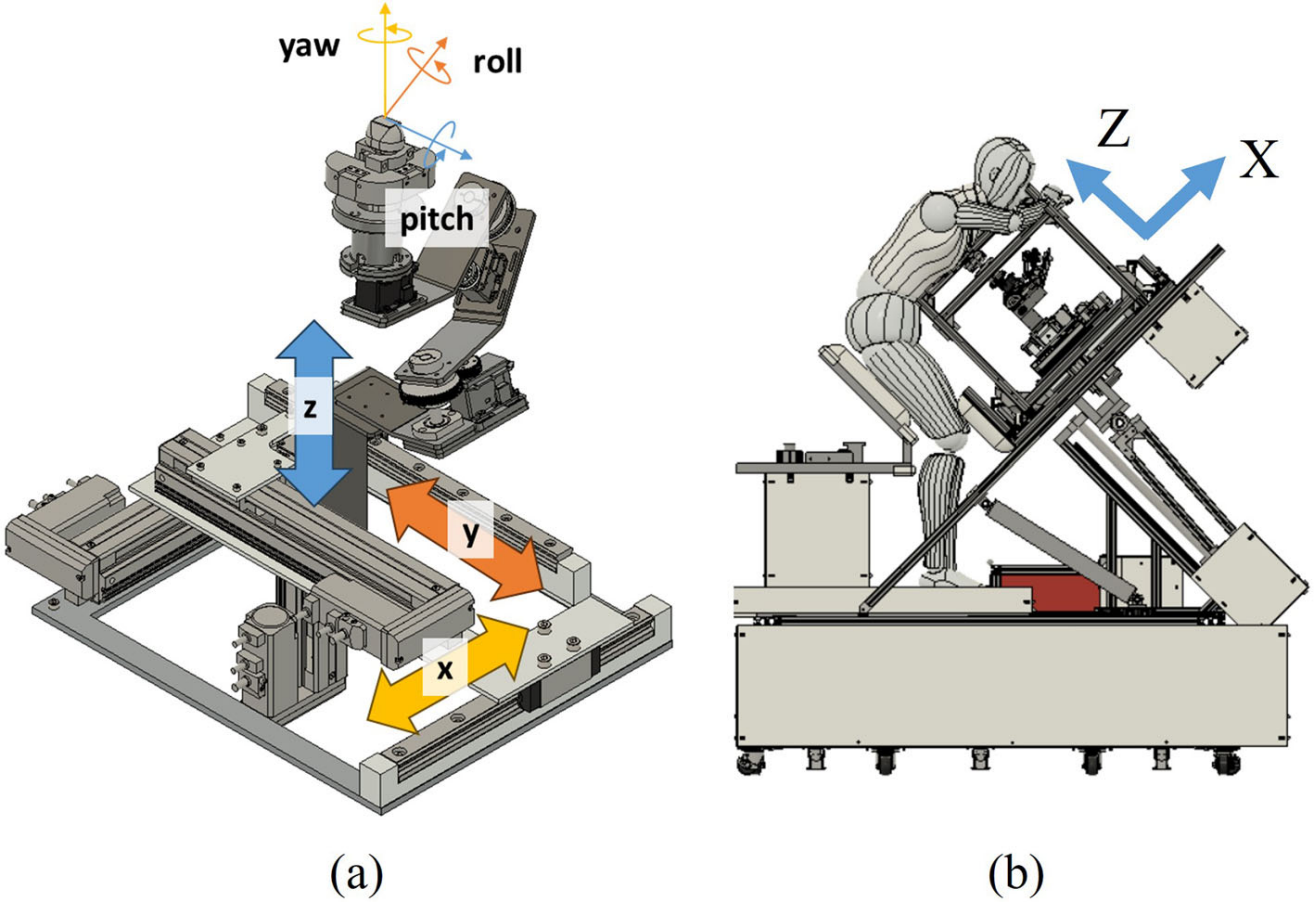
Degrees of freedom of the unit/positioning with the patient. **a** Degrees of freedom of the PSU. **b** Positioning of the PSU and the patient

The proposed method primarily comprises (i) an optimized search behavior generation algorithm and (ii) an optimized path generation algorithm (Fig. 2). Here, the optimized search behavior generation algorithm (i) takes as input the detection confidence C of the heart component detected in the US image obtained by the echocardiographic robot and the position (X, Y) of the probe when obtaining the US image. This algorithm outputs the next position to be searched by the probe using DQN. The optimized path generation algorithm (ii) uses the information on the next location to be searched by the robot calculated by algorithm (i) as input and generates a path that enables the robot to search in the shortest possible time by processing, e.g., by avoiding previously searched locations. These algorithms are designed to find the optimal solution quickly in the presence of local solutions, which were described in the previous section. These algorithms are described in detail in the next section. Note that the probe position and angle were adjusted using a probe scanning unit (PSU) consisting of a serial link mechanism with three degrees of freedom for rotation (roll, pitch, and yaw) and a linear motion mechanism with three degrees of freedom for translation (X, Y, and Z) (Fig. 3a). The positional relationship between the PSU and the patient is shown in Fig. 3b. Also, since the PSU is equipped with a function that adjusts the Z-axis position according to the unevenness of the body surface so that the probe can always be pressed against the body surface, the proposed method in this paper aims to derive the X and Y coordinates of the probe that can illustrate the heart component based on the assumption that this function is installed.

### Optimized robotic navigation system for heart components

#### Optimized search behavior generation algorithm

**Fig. 4:**
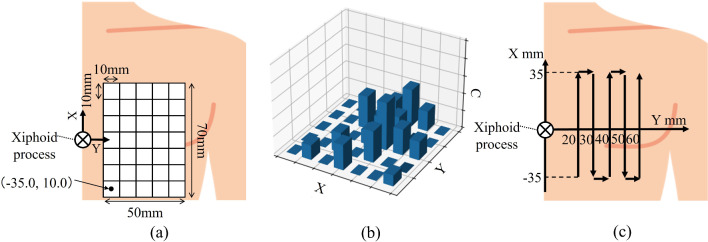
DQN learning conditions: **a** grid world on the chest plane, **b** XYC state space, and **c** five-way traveling scan path

The optimized search behavior generation algorithm uses DQN to search for heart components at time costs that are similar to those of physicians. This method also avoids local solutions using an epsilon greedy algorithm and experience replay [9] in learning DQN and by devising reward conditions. The structure of the DQN model, agent, environment, behavior, state, and reward conditions to solve the heart component search behavior optimization problem using deep reinforcement learning are described as follows.

(A) *Structure of the DQN model*: The number of nodes in the hidden layer of the DQN model used in this method is 100. The number of hidden layers, D, on the other hand, is a variable and is determined by experimentation, as described in the next section.
(B) *Agent*: The agent is the tip position of the probe moved by the echocardiography robot. The coordinate system, as shown in Fig. 4a, is set with the xiphoid process of the human body as the origin, the vertical axis as the X-axis, and the forehead axis as the Y-axis. Note that the probe angle was fixed at 0° for roll, pitch, and yaw, respectively.
(C) *Environment*: The environment in which the probe tip position (i.e., the agent) can act is the X–Y grid world on the chest plane, as shown in Fig. 4a.
(D) *Behavior*: The behavior that can be performed on the grid world of the probe tip position as the agent includes nine types of movements in eight directions (up, down, left, right, and diagonal) and stops.
(E) *State*: The state in which the probe tip position as the agent is observable on the grid world (Fig. 4b**)** is defined as the XYC state space comprising the probe positions X and Y (grid numbers) and the object detection confidence C of the heart component in the US image acquired by the probe at each position. The object detection confidence C of the heart component in each grid is calculated through the following steps: (i) during the search process, the object detection confidence of the heart component is calculated for each ultrasound image acquired in the grid using the object detection model Yolo v8. (ii) The median value of the object detection confidences of the heart component calculated in (i) is selected as the object detection confidence C of the heart component in the grid. The initial position of the probe is assumed to start at (X_s_, Y_s_) in the grid map. This X_s_, Y_s_ is a variable and is determined by experimentation as described in the next section.
F) *Reward conditions*: The reward conditions are defined as shown in Table 1. The reward conditions can be divided into two categories, i.e., (i) a condition to reduce the search time and (ii) a condition to avoid local solutions. The first three items in Table 1 belong to (i) and the last four items in Table 1 belong to (ii). The following is a detailed description of the two distinctive items in this section that address the issues in this paper.

**Table 1:** DQN reward conditions

Reward conditions	Score
Perform one move	− 0.1
Move out of search range	− 0.3
Stop	− 3.0
Stops at the optimal solution position after less than 35 moves	20.0
Change in C at the destination by Δ from the previous position C when moving	10.0・Δ
Stops at the optimal solution	5.0
Stops at a position other than the optimal solution	− mL・(Cm-Cp)

First, let *C*_*b*_ be the heart component confidence score at the point before the move for a single move and *C*_*a*_ be the heart component confidence score at the point after the move for a single move, then the calculated change in heart component confidence score when the move is made, Δ, is calculated by Eq. 1.

1 $$ \begin{array}{*{20}c} {\Delta = C_{a} - C_{b} } \\ \end{array} $$

Here, 10.0•Δ points are added or subtracted for each Δ change in the heart component confidence C per move to encourage movement in the direction of increasing C.

Next, when the probe stops a nonoptimal solution, the points P_ld_ shown in Eq. 2 are deducted from the reward. Note that m_L_ is the variable, *C*_*m*_ is the confidence score of heart components at the optimal solution, and *Cp* is the confidence score of heart components at the stopping position.

2 $$ \begin{array}{*{20}c} {P_{ld} = m_{L} \left( {C_{m} - C_{p} } \right)} \\ \end{array} $$

These reward conditions allow the system to move when the total reward can be expected to increase by moving further in a certain state, i.e., when the current position is a nonoptimal solution, and to suppress the stopping behavior at nonoptimal solution positions, including local solutions.

*D*, *m*_*L*_, *X*_*s*_, and *Y*_*s*_ are variables and are determined by experimentation as described in the next section. The advantages and disadvantages of increasing values of *D*, *m*_*L*_ are shown in Table 2. It is believed that the *D*, *m*_*L*_ appropriate for the complexity of the present task and the characteristics of the local solution will be determined experimentally. As for the initial position of the probe *X*_*s*_, *Y*_*s*_, it is considered that the position where the heart component is most likely to be located relative to the body’s xiphoid process, which is the origin of the grid map, will be determined experimentally.

**Table 2:** The advantages and disadvantages of increasing values of D, m_L_

Parameter	Advantage	Disadvantage
D	Enabling the classification of complex optimal behaviors	Occurrence of overfitting
mL	Improvement in the performance of avoiding local solutions	Increase in the probability of passing the optimal solution

#### Optimized path generation algorithm

**Fig. 5:**
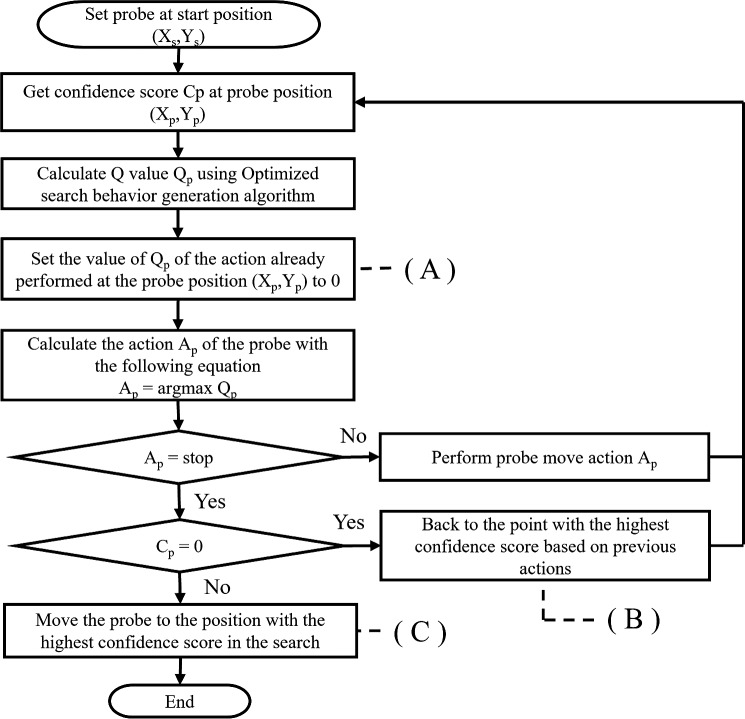
Flowchart of optimized path generation algorithm

Path generation using only the information on the optimal action calculated by the optimized search behavior generation algorithm has the following problems: (i) it repeatedly searches for a position once passed, which results in an infinite number of moves without selecting a stopping action; (ii) it stops at a grid with zero mitral valve confidence; and (iii) if an optimal solution is found during the search, it is impossible to return to the optimal solution position. Thus, the optimized path generation algorithm is designed to solve the above issues using information on the DQN’s action value vector as the main axis of the algorithm. The optimized path generation algorithm is implemented as explained in Fig. 5.

In (A) of Fig. 5, when the probe comes to the same position two or more times, it chooses the best action among those other than the search action already chosen at that position. This avoids problem (i). In (B) of Fig. 5, when the selected search action is a stop action and the confidence C of the heart component at the current probe position is 0, the probe returns to the probe position where the confidence C of the heart component on the trajectory searched to this point is the maximum and begin again from this position. This avoids problem (ii). In (C) of Fig. 5, when the selected search action is a stop action, the search is terminated, and the position with the highest heart component confidence C on the trajectory searched so far is estimated as the position where the heart component can be obtained most clearly. This avoids problem (iii).

## Experimental setup

The proposed optimized robotic navigation system for heart components was validated by (i) constructing and evaluating the optimized search behavior generation algorithm and (ii) conducting a performance evaluation of the proposed system. The evaluation experiments were conducted using a seated echocardiographic robot [5], a matrix array sector probe (X5-1, Philips, The Netherlands), and US equipment (EPIQ 7G, Philips, The Netherlands). The subjects included 24 males in their teens to twenties (height: 168.5 ± 8.5 cm; weight: 65 ± 17 kg). Note that the mitral valve, which is a fundamental element in the automatic parasternal long-axis view search [10] in the echocardiography robot, was used as an example of a heart component to be searched. The calculation of mitral valve confidence was derived using the Yolo v8 deep learning model, which has been used previously in automated long-axis view search using an echocardiography robot. The confidence score of the mitral valve is calculated using the Yolo v8 model trained with 300 epochs and 8 batch sizes using 7422 images (5938 for training and 1484 for validation) annotated with the mitral valve according to the rule that the mitral valve is not fully closed, as defined by the authors. The mean average precision 50 (mAP50) of this mitral valve object detection model was 0.773.

### Construction and evaluation of optimized search behavior generation algorithm

The validity of using DQN to search for heart components in transthoracic echocardiography was evaluated by deriving the parameters of a search optimal action generation algorithm and analyzing its accuracy. This process involved two stages, i.e., dataset creation and parameter derivation and evaluation based on training using the data. The evaluation method is described in detail in the following.

A) Dataset creation
  1. The probe of the echocardiography robot is moved to the origin.
  2. The subject is seated on the echocardiography robot, and the subject's xiphoid process is placed against the tip of the probe.
  3. The probe is scanned at approximately 10 mm/s along the five-way traveling scan path shown in Fig. 4c. The breath instruction proposed in the literature [11] is provided during the traverse to minimize the effect of missing lungs in the acquired US images.
  4. The mitral valve position is estimated using a previously proposed algorithm [10] after completing the five-way traveling scan. US images are acquired after the probe tip position is moved to the estimated position.
  5. The acquired US images are observed manually. Here, if the mitral valve is acquired clearly, the group of US images acquired at the five-way traveling scan is added to the dataset.
B) Derivation and evaluation of parameters for optimized search behavior generation algorithm. The following table shows the method used to determine the depth of the hidden layer D (Fig. 2), the reduction factor mL for mitral loss of confidence at a stop, and the probe search starting position *X*_*s*_ and *Y*_*s*_ (Fig. 2) in this system. In this evaluation, three-part cross-validation (by subject) was performed on a dataset with 316 samples. Three-part cross-validation is a method in which training and test data are separated at a ratio of 2:1 and alternately trained and evaluated, which is a learning method with less overfitting.
  1. The system was trained with *m*_*L*_ fixed at 10, *X*_*s*_ and *Y*_*s*_ for each of the 35 grids shown in Fig. 4a, and D was changed to 2, 4, and 6. The accuracy and efficiency of each D were then derived to determine the D value to be used in the system.
  2. The system was trained by fixing D to the value determined in step (1), changing *X*_*s*_ and *Y*_*s*_ for each of the 35 grids shown in Fig. 4a, and changing *m*_*L*_ to 10, 20, and 40. The accuracy and efficiency of each *m*_*L*_ were derived to determine the *m*_*L*_ to be used in the system.
  3. The system was trained by fixing D to the value determined in step (1) and *m*_*L*_ to the value determined in step (2), and then changing *X*_*s*_ and*Y*_*s*_ to the grid of 35 shown in Fig. 4a. The accuracy and efficiency of each combination of *X*_*s*_ and *Y*_*s*_ were derived to determine the *X*_*s*_ and *Y*_*s*_ values to be used in this system.
  4. The characteristics and validity of the optimized search behavior generation algorithm were evaluated based on the results of steps (1) to (3). The accuracy and efficiency of the proposed algorithm were evaluated using the Cohen's effect size with respect to the conventional method, with the effect size *d*_*C*_ regarding the mitral valve confidence as the accuracy criterion and the effect size *d*_*C*_ regarding the number of search actions as the efficiency criterion. Let $\overline{{X }_{pc}}$ and $\overline{{X }_{cc}}$ be the mean values of the mitral valve confidence scores calculated by the proposed and conventional methods, respectively, and let *S*_*c*_ be the standard deviation between these two groups of data. Then, *d*_*C*_ becomes **Eq. **3. Let $\overline{{X }_{pa}}$ and $\overline{{X }_{ca}}$ be the mean values of the number of search actions calculated by the proposed and conventional methods, respectively, and let *s*_*a*_ be the standard deviation between these two groups of data. Then, *d*_*A*_ becomes **Eq. **4.
    3 $$ \begin{array}{*{20}c} {d_{C} = \frac{{\left| {\overline{{X_{pc} }} - \overline{{X_{cc} }} } \right|}}{{S_{c} }}} \\ \end{array} $$
    4 $$ \begin{array}{*{20}c} {d_{A} = \frac{{\left| {\overline{{X_{pa} }} - \overline{{X_{ca} }} } \right|}}{{S_{a} }}} \\ \end{array} $$

Note that the conventional method is to estimate the location of the mitral valve as the position where the US image with the highest confidence score of the mitral valve was acquired from a group of US images of the entire chest acquired by performing the five-way traveling scan as shown in Fig.4c**.** In this experimental study, when *d*_*C*_ was less than 0.5, the accuracy of the proposed method decreased only slightly compared to the conventional method, and it was evaluated that the accuracy of the proposed method was maintained. When *d*_*A*_ is greater than 0.8, the efficiency of the proposed method is evaluated to be higher than that of the conventional method [12].

### Performance evaluation of optimized robotic navigation system for heart components

We evaluated the validity of the proposed optimized robotic navigation system for heart components by conducting a search in a simulation space and analyzing the results to determine whether the proposed system can perform inspections in a shorter time while maintaining sufficient search accuracy compared to conventional methods. The details of the evaluation method are described as follows.

1. The 316 datasets obtained in the previously mentioned section of the construction and evaluation of the optimized search behavior generation algorithm are divided into three groups A, B, and C.
2. Using parameters determined during the validation of the construction and evaluation of the optimized search behavior generation algorithm, DQN models are constructed: model *α* with data sets A and B, model *β* with data sets B and C, and model *γ* with data sets A and C.
3. Data sets A, B, and C were searched by optimized robotic navigation systems with models *β*, *γ*, and *α*, respectively. During these searches, the confidence score of the mitral valve at the time of stopping and the number of actions until stopping were calculated.
4. The validity of the optimized robotic navigation system was evaluated using the mitral valve confidence at a stop as the accuracy of the system and the number of moves as the system’s efficiency.
5. The mitral valve characteristics in the US images obtained at the time of the stop and the loss of confidence rate of the mitral valve *L*_*c*_ (**Eq. **5) were analyzed.
  5 $$ \begin{array}{*{20}c} {L_{C} = 1 - \frac{{C_{S} }}{{C_{m} }}} \\ \end{array} $$

Here, *C*_*s*_ denotes the mitral valve confidence at the time of stopping, and *C*_*m*_ denotes the mitral valve confidence at the optimal solution position. *L*_*c*_ is a measure of the quality of the US images obtained from the estimated probe position in the proposed method compared to the optimal solution. The evaluation of the quality of the US images when estimating a nonoptimal solution in the proposed method is based on the distribution of *L*_*c*_ and the relationship of the visibility of heart components in the US image to the value of *L*_*c*_.

## Evaluation

### Results

#### Construction and evaluation of optimized search behavior generation algorithm

**Fig. 6:**
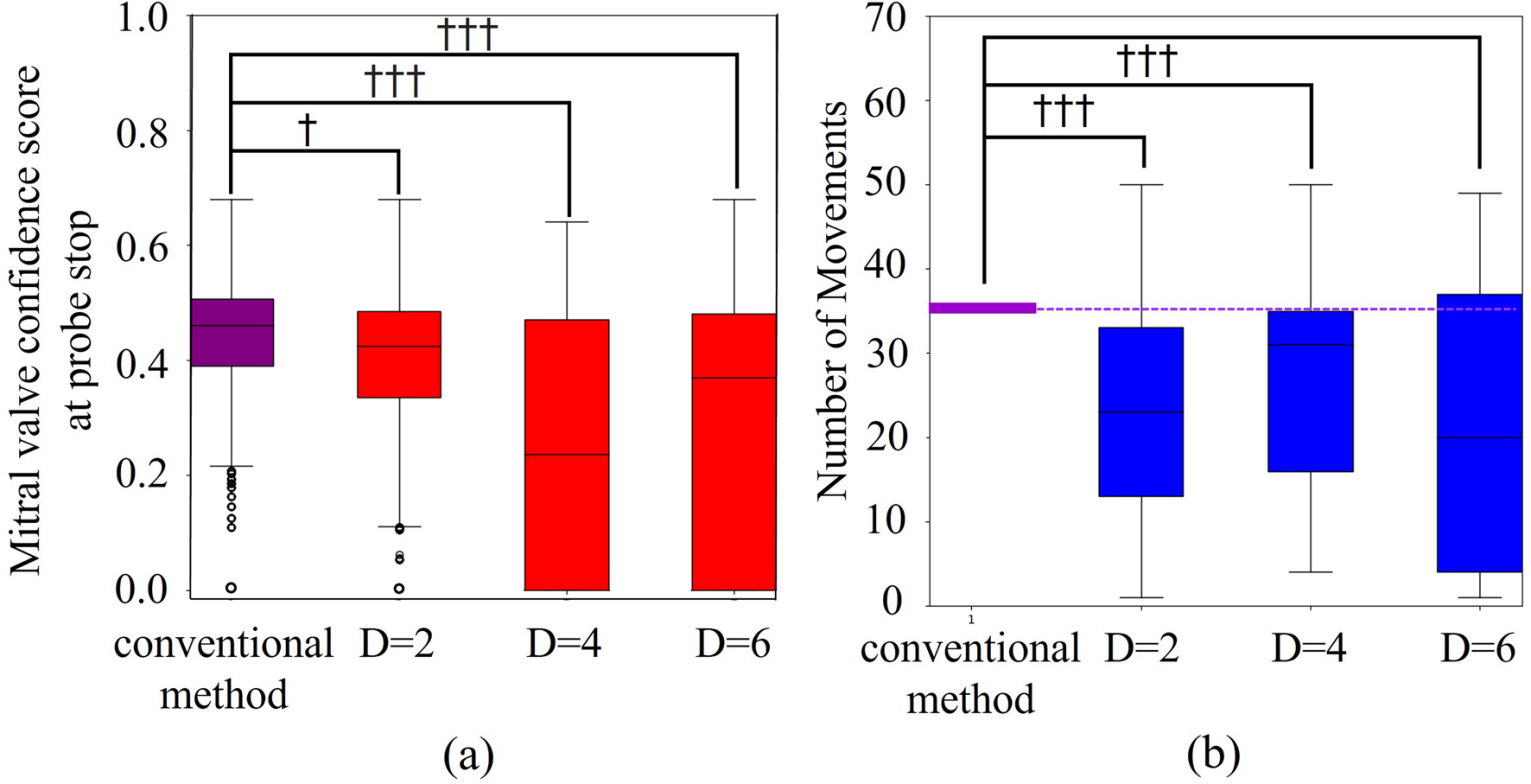
Relationship between the value of D and **a** the mitral valve confidence and **b** the total number of actions

The relationship of mitral valve confidence and total number of actions relative to the value of D is shown in Fig. 6. The effect size of *d*_*C*_ was 0.47, 1.13, and 0.85, and the effect size of *d*_*A*_ was 1.38, 0.94, and 1.31 for *D* = 2, 4, and 6, respectively. When *D* was set to 2, the proposed system exhibited a small reduction in accuracy and a large increase in efficiency; thus, *D* was set to 2.

**Fig. 7:**
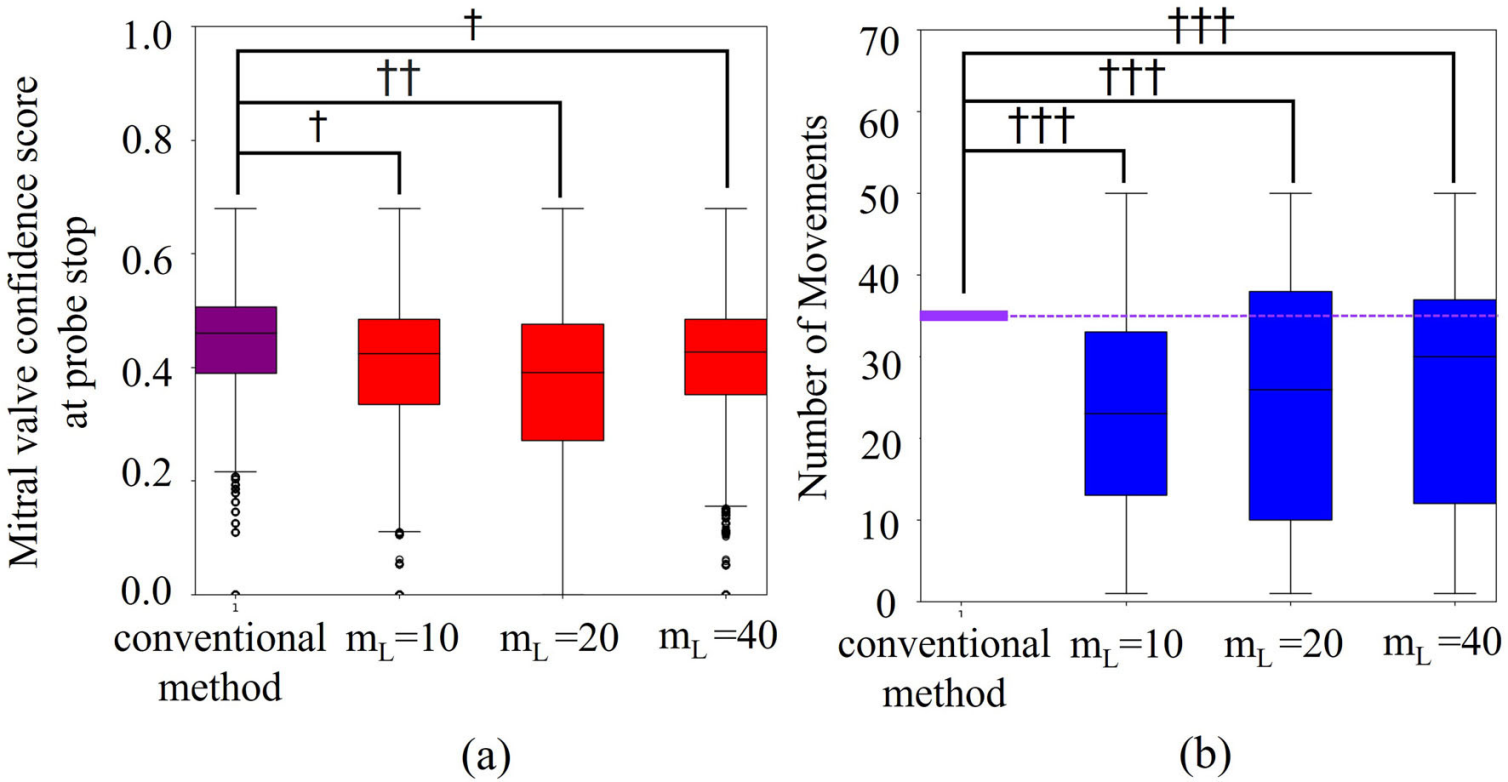
Relationship between the value of m_L_ and **a** the mitral valve confidence, **b** and the total number of actions

**Fig. 8:**
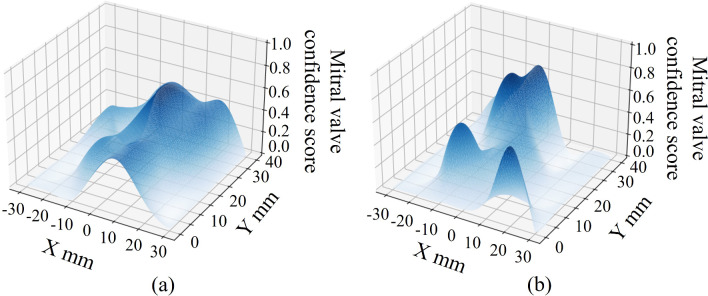
Examples of confidence maps of the subject’s mitral valve: **a** Higher accuracy when mL is 10 compared to when mL is 40, **b** Higher accuracy when mL is 40 compared to when mL is 10

The relationship of mitral valve confidence and total number of actions relative to the value of m_L_ is shown in Fig. 7**.** The effect size of *d*_*C*_ was 0.45, 0.66, and 0.34, and the effect size of *d*_*A*_ was 1.38, 1.06, and 1.02 for *m*_*L*_ = 10, 20, and 40, respectively. The accuracy of *mL* = 10 and *mL* = 40 exhibited a small reduction in accuracy and a large increase in efficiency. Figure 8a shows an example of a confidence map of the mitral valve of the subject with higher accuracy when *m*_*L*_ is 10 compared to when *m*_*L*_ is 40, and Fig. 8b shows an example of a confidence map of the mitral valve of the subject with higher accuracy when *m*_*L*_ is 40 compared to when *m*_*L*_ is 10. The highest accuracy, i.e., *mL* = 40, was used in this system because accuracy is more important than efficiency for health care systems.

**Fig. 9:**
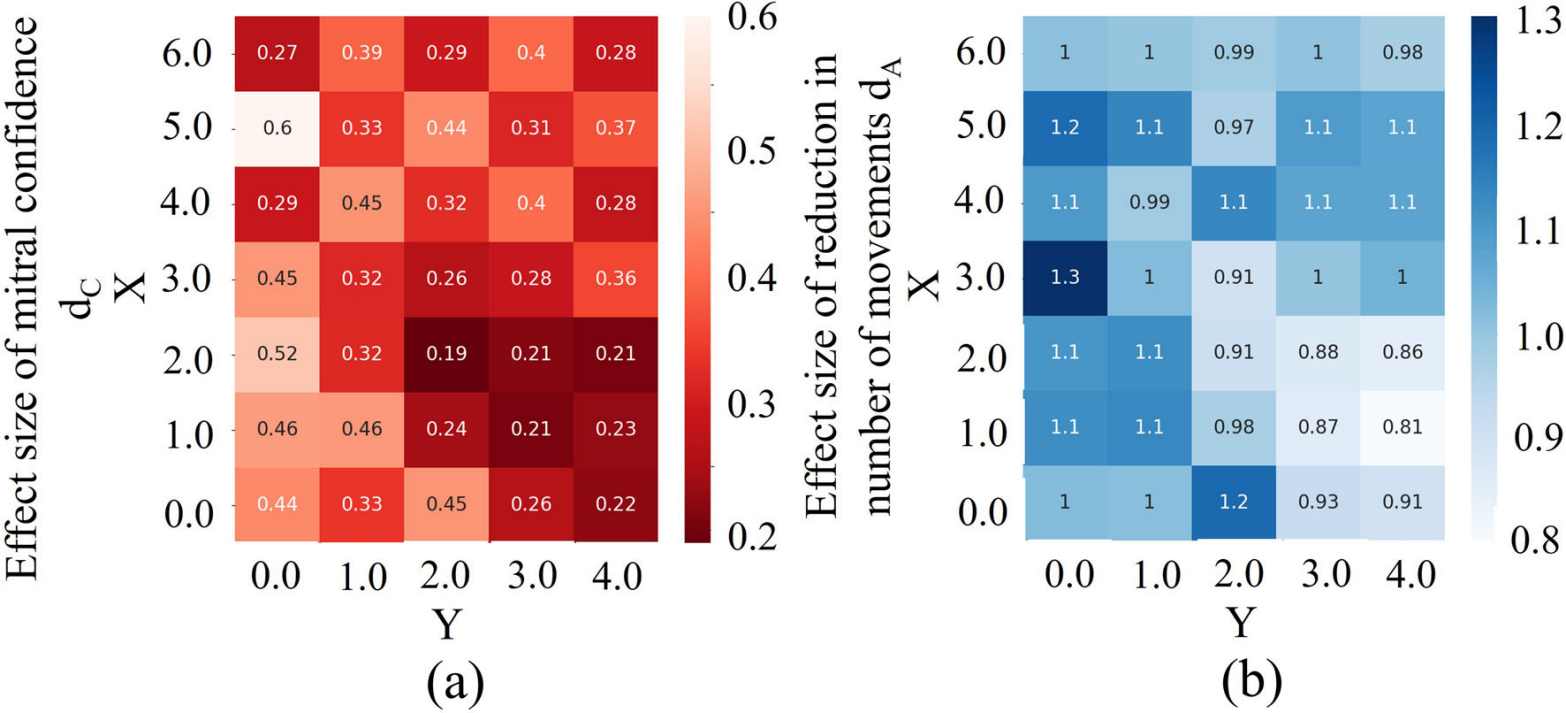
Relationship between the search start position and **a** the mitral valve confidence and **b** the total number of actions

The relationship of mitral valve confidence and total number of actions relative to the value of search start position *X*_*s*_, *Y*_*s*_ is shown in Fig. 9. The effect size of *d*_*C*_ was less than 0.5 except for some starting positions, indicating that the search accuracy was maintained with respect to the conventional method. In particular, *d*_*C*_ was 0.19 at (*X*_*s*_, *Y*_*s*_) = (2, 2), which was the highest search accuracy. On the other hand, the effect size of *d*_*A*_ was larger than 0.8 at all starting positions, indicating higher efficiency than the conventional method. In particular, *d*_*A*_ was 1.3 at (*X*_*s*_, *Y*_*s*_) = (3, 0), which is the highest efficiency. These results show that (*X*_*s*_, *Y*_*s*_) = (2, 2) has the highest accuracy and search efficiency compared to conventional methods, so this position was used in this system because accuracy is more important in healthcare systems.

#### Performance evaluation of optimized robotic navigation system for heart components

**Fig. 10:**
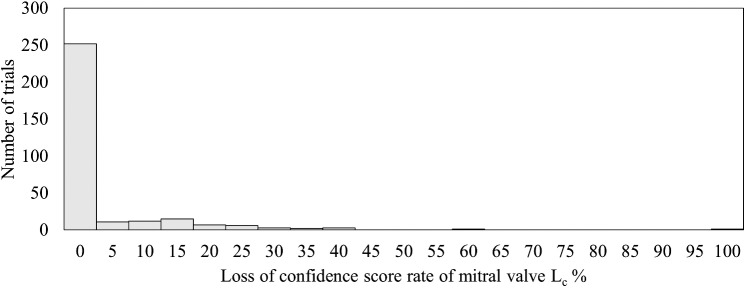
The distribution of the loss of the confidence rate of the mitral valve *L*_*c*_

**Fig. 11:**
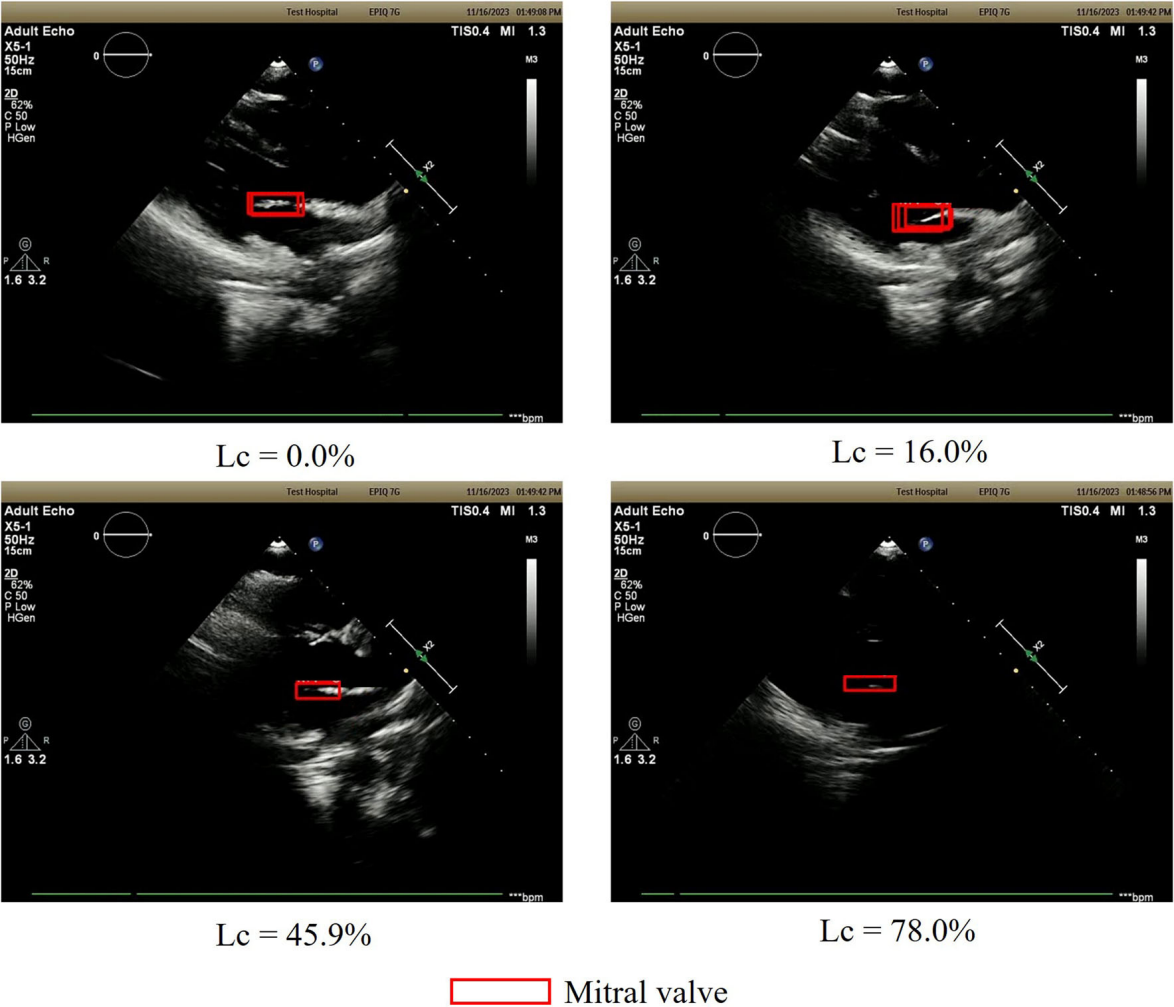
The relationship of the visibility of heart components in the US image to the value of L_c_

The results of this evaluation are shown in Table 3**.** The average inspection time for all trials was 48.6 s, representing 56.6% of the average inspection time of the conventional method (85.9 s). The distribution of the loss of the confidence rate of the mitral valve *L*_*c*_ is shown in Fig. 10. Also, the relationship of the visibility of heart components in the US image to the value of *L*_*c*_ is shown in Fig. 11. As can be seen, *Lc* was distributed in the low range, with a mean value of 16.3%. Note that *C*_*m*_ used in Table 2 has already been defined in methods optimized robotic navigation system for heart components section and represents the confidence score of heart components at the optimal solution.

**Table 3:** Results of the performance evaluation of optimized robotic navigation system

		Number of movements		Total
		Less than 35	35 or more	
Mitral valve confidence score at prove stop	*C* = *C*_*m*_	38.9%	35.4%	74.4%
	0.0 < *C* < *C*_*m*_	23.1%	2.2%	25.3%
	*C* = 0.0	0.3%	0.0%	0.3%
Total		62.3%	37.7%	100.0%

## Discussions

The results of the validation experiment for construction and evaluation of optimized search behavior generation algorithm indicate that the proposed optimized search behavior generation algorithm realizes highly efficient mitral valve search while maintaining sufficient accuracy. The fact that accuracy and efficiency were maximal at D = 2 suggests that even a small number of hidden layers is sufficient model representation for the task of searching for probe positions that enable clear acquisition of the mitral valve. Conversely, the fact that accuracy and efficiency decreased at D = 4 and 6 suggests that overlearning is occurring due to excessively complex model representation. This indicates that despite individual subject differences, there is optimized behavior shared across subjects. Thus, the proposed method is applicable to a wide range of patients. High accuracy was observed when m_L_ is 10 or 40. As m_L_ increases, it becomes easier to avoid local solutions, but the probability of passing the optimization solution increases (see Table 2). Thus, when there are few local solutions, as in Fig. 8a, it is more accurate to have a small m_L_ and not pass through the optimized solution when it is reached, and to be able to estimate its location as the optimized solution. When there are many local solutions, as in Fig. 8b, the accuracy is improved if m_L_ is large, and the entire chest is searched to reach the optimized solution without stopping at the position of the local solution. We observed a tendency for accuracy to be higher when the search was initiated from a low position on the lateral side of the body. This is considered to be due to the presence of the sternal body and lungs in the center of the body, which interfere with US image acquisition. Note that the heart is located toward the lower left of the body. The low position on the lateral side of the body provides a large area where the heart can be observed without being obstructed by the sternal body and lungs, thereby making it easier to search for the heart components.

The results in Table 3 show three main outcomes when the proposed method is applied: (i) the optimal solution is achieved with fewer actions than the conventional method, (ii) the optimal solution is achieved with more actions than the conventional method, and (iii) the nonoptimal solution is achieved with fewer actions than the conventional method. For (iii), Fig. 10 indicates that the loss of confidence rate of the mitral valve (*Lc*) is low even in the nonoptimal solution, and Fig. 11 shows that the shape of the entire mitral valve can be read off when *Lc* is low. This can be considered that in case (iii), the estimated position of the heart component is near the optimal solution, allowing clear observation of the heart component. Thus, while the proposed method may not always efficiently estimate the optimal solution as the hypothesis described in the Introduction, it can effectively estimate a position where cardiac components are clearly visible. However, in case (iii), the number of actions increases due to a large mL value used to avoid local solutions. Future work should focus on proposing more efficient search methods using appropriate mL values based on factors like individual body shape. Based on the results of this verification, it is believed that the proposed method can be implemented in the real world to reduce inspection time by approximately 50.0 s while ensuring accuracy. On the other hand, the verification in this study was conducted in a simulation and not on an actual robot. Therefore, when the proposed method is implemented on an actual robot, some images may not be clearly acquired when the probe is scanned over the chest, which may cause problems in the calculation of the optimal action of the proposed method. It is necessary to introduce and verify the proposed method on an echocardiography robot to see if these problems occur, and if so, to devise a method to enable the acquisition of clear US images at all times and to consider using the proposed method in combination with this method.

## Limitations

There are several limitations in the proposed method and the process used to validate the method. First, the proposed method was applied to only male subjects in their teens and twenties. Thus, the proposed method was not validated for a wide range of patient ages and genders. Therefore, in the future, it will be necessary to perform this verification with subjects of diverse ages and different genders to determine whether the proposed method is generalizable. Also, the number of datasets is not known whether it was sufficient. In the future, it is necessary to verify whether the number of data sets used in this study was sufficient by increasing the number of data sets and comparing the accuracy of the trained model with that of the model in this paper.

Second, in the proposed method, the roll, pitch, and yaw angles of the probe are fixed at 0°. This is based on the search method proposed in Ref. 7, in which the roll, pitch, and yaw angles of the probe are fixed at 0° and the probe angle is adjusted after searching for a probe position that satisfies the conditions. However, it is possible that the probe angle may need to be adjusted when US images become blurred due to the influence of bone or other factors. In the future, it will be necessary to construct a search algorithm that takes probe rotation into consideration.

Finally, the proposed method assumes that the patient does not move while on the robot, but the patient may move during the examination, causing the positional relationship between the robot and the patient to shift, which would reduce the accuracy of the proposed method. In the future, it will be necessary to introduce a function that measures the patient’s movement during the examination and corrects the probe position according to the patient’s movement, and combine this function with the proposed method.

## Conclusion

The purpose of this study was to develop a system to optimize robotic navigation of heart components using a transthoracic echocardiography robot. In transthoracic echocardiography, in the search for heart components, local solutions are caused by factors that obscure US images, e.g., the ribs, lungs, and fat. To address this issue, the proposed method introduces an optimized search behavior generation algorithm that avoids multiple local solutions and searches for the optimal solution. In addition, the proposed method includes an optimized path generation algorithm that minimizes the search path and enables a short search time. The accuracy and efficiency of the proposed method were verified experimentally. Here, the optimized solution attainment rate was 74.4%, and the mitral valve confidence loss rate at nonoptimized solution stops was suppressed to 16.3% on average. The average estimated inspection time was 48.6 s, representing 56.6% of the inspection time of the conventional method, which suggests the usefulness of the proposed method. In the future, we plan to integrate the proposed method with a basic view search method using a practical transthoracic echocardiography robot to realize fast and automated echocardiographic examinations.

## References

[1] WHO (World Health Organization) (2021) Cardiovascular diseases (CVDs). World Health Organization.https://www.who.int/news-room/fact-sheets/detail/cardiovascular-diseases-(cvds). Accessed 9 November 2022

[2] Fang TY, Zhang HK, Finocchi R, Taylor RH, Boctor EM (2017) Force-assisted ultrasound imaging system through dual force sensing and admittance robot control. Int J Comput Assist Radiol Surg 12(6):983–991. 10.1007/s11548-017-1566-9 28343302 10.1007/s11548-017-1566-9

[3] Esteban J, Simson W, Requena Witzig S, Rienmüller A, Virga S, Frisch B, Zettinig O, Sakara D, Ryang YM, Navab N, Hennersperger C (2018) Robotic ultrasound-guided facet joint insertion. Int J Comput Assist Radiol Surg 13(6):895–904. 10.1007/s11548-018-1759-x 29671200 10.1007/s11548-018-1759-x

[4] Shida Y, Tsumura R, Watanabe T, Iwata H (2021) Heart position estimation based on bone distribution toward autonomous robotic fetal ultrasonography. 2021 IEEE International Conference on Robotics and Automation, no.940 10.1109/ICRA48506.2021.9560839

[5] Shida Y, Sugawara M, Tsumura R, Chiba H, Uejima T, Iwata H (2023) Diagnostic posture control system for seated-style echocardiography robot. Int J CARS 18:887–897. 10.1007/s11548-022-02829-3 10.1007/s11548-022-02829-3 PMC10113306 36881353

[6] Hase H, Azampour M F, Tirindelli M, Paschali M, Simson W, Fatemizadeh E, Navab N (2021) Ultrasound-guided robotic navigation with deep reinforcement learning, 2020 IEEE/RSJ International Conference on Intelligent Robots and Systems (IROS), 10.1109/IROS45743.2020.9340913

[7] Li K, Li A, Xu Y, Xiong H, Meng MQH (2023) RL-TEE: autonomous probe guidance for transesophageal echocardiography based on attention-augmented deep reinforcement learning. IEEE Trans Autom Sci Eng. 10.1109/TASE.2023.3246089

[8] Milletari F, Birodkar V, Sofka M (2019) Straight to the point: reinforcement learning for user guidance in ultrasound. Smart Ultrasound Imaging Perinat Preterm Paediatr Image Anal. 10.1007/978-3-030-32875-7_1

[9] Lin LJ (1992) Self-improving reactive agents based on reinforcement learning, planning and teaching”. Mach Learn 8:293–321. 10.1007/BF00992699

[10] Shida Y, Kumagai S, Tsumura R, Iwata H (2023) Automated image acquisition of parasternal long-axis view with robotic echocardiography. IEEE Robot Autom Lett 8(8):5228–5235. 10.1109/LRA.2023.3292568

[11] Hashimoto T, Shida Y, Kumagai S, Iwata H (2023) Development of a seated echocardiography robot - lung volume and body posture conditions that enable clear mitral valve echocardiographic images. 2023 IEEE/SICE Int Symp Syst Integr. 10.1109/SII55687.2023.10039203

[12] Cohen J (1962) Statistical power analysis for the behavioral sciences, 1st edn. Academic Press, Cambridge

